# Practical Notes on Diagnosis and Treatment

**Published:** 1911-02-04

**Authors:** 


### Practical Notes on Diagnosis and Treatment.

### Opium and Bright's Disease.

Morphine and opium are often said to be contra-indicated in Bright's disease. This seems to be due to the belief that morphine is excreted by the kidney, which has now been shown to be erroneous. There seems to be no reason to believe that morphine is harmful in these conditions, and in some forms of uræmia it has even been of considerable benefit.—*Professor Cushny.*

### The Preservation of Drugs and Instruments in the Tropics.

Bombay, Colombo, and Singapore are cited as typical of the worst localities, where heat, light, and moisture combine to produce the most destructive effects upon all organic substances. Vegetable drugs in the form of leaves and roots, although compressed tightly in bales, cannot be stored for many weeks during the monsoon without being attacked by fungi, which destroy the parts affected. Deliquescent salts, by the absorption of moisture, alter their weight value, and compounded drugs in time lose their value by the chemical interaction of their constituents. The penetration of moisture into closed vessels is due to two principal causes—namely, the daily fluctuation of temperature (equal to 15° F.) and changes of barometric pressure, which take place twice daily. Figures have been given showing how expansion and contraction and humidity of the air in the containing vessels are affected by these two influences. Water vapour is thus carried continuously in and out of imperfectly closed vessels, and the evil effects are most apparent in vessels that are not full. The various modes of closing bottles and other vessels with wax, paraffin-saturated cork, rubber rings or washers, are all affected by changes of volume, temperature, or humidity of the air, that swell, crack, or decompose them. Absolute dryness of the air of a container is impracticable, but it has been shown how, by means of tightly fitting lids, adequate soldering of tin boxes, proper building of wooden boxes, etc., all materials may be preserved in good condition for a certain time in an atmosphere containing some moisture. Bags of quicklime may be placed in the interior of boxes, etc., to keep the air dry for the protection of books, papers, instruments, etc. Protection against the white ant may be obtained by painting the exterior of each box with mineral spindle oil, which is worth about 1s. 8d. per gallon. This oil sinks into the wood and imparts such a bitter, unpleasant taste to it that vermin avoid it. Leather, velvet, and cloth linings are condemned for instrument cases on account of their liability to absorb moisture. Next to plated metal the best material for cases appears to be wood that has been soaked in melted hard paraffin and stoved until the surplus drains off.

